# Cost-effectiveness and accuracy of cervical cancer screening with a high-risk HPV genotyping assay vs a nongenotyping assay in China: an observational cohort study

**DOI:** 10.1186/s12935-020-01512-4

**Published:** 2020-08-28

**Authors:** Binhua Dong, Lihua Chen, Wenyu Lin, Yingying Su, Xiaodan Mao, Diling Pan, Guanyu Ruan, Huifeng Xue, Yafang Kang, Pengming Sun

**Affiliations:** 1 Department of Gynecology, Laboratory of Gynecologic Oncology, Fujian Maternity and Child Health Hospital, Affiliated Hospital of Fujian Medical University, 18 Daoshan Road, Fuzhou, 350001 Fujian People’s Republic of China; 2 Fujian Key Laboratory of Women and Children’s Critical Diseases Research, Fujian Maternity and Child Health Hospital, Affiliated Hospital of Fujian Medical University, Fuzhou, 350001 Fujian People’s Republic of China; 3 State Key Laboratory of Molecular Vaccinology and Molecular Diagnostics, National Institute of Diagnostics and Vaccine Development in Infectious Diseases, Strait Collaborative Innovation Center of Biomedicine and Pharmaceutics, School of Public Health, Xiamen University, Xiamen, Fujian China; 4 Department of Pathology, Fujian Maternity and Child Health Hospital, Affiliated Hospital of Fujian Medical University, Fuzhou, 350001 Fujian People’s Republic of China; 5 Fujian Provincial Cervical Disease Diagnosis and Treatment Health Center, Fujian Maternity and Child Health Hospital, Affiliated Hospital of Fujian Medical University, Fuzhou, 350001 Fujian People’s Republic of China

**Keywords:** Human papillomavirus, Genotyping, Cervical cancer, Cancer screens, Cotesting

## Abstract

**Background:**

New screening techniques may affect the optimal approaches for the prevention of cervical cancer. We evaluated the cost-effectiveness and accuracy of alternative screening strategies to provide evidence for cervical cancer screening guidelines in China.

**Methods:**

In total, 32,306 women were enrolled. The current screening with Cervista^®^ high-risk human papillomavirus (HR-HPV) nongenotyping and cytology cotesting (Cervista^®^ cotesting) was compared with PCR-reverse dot blot HR-HPV genotyping and cytology cotesting (PCR-RDB cotesting). All eligible participants were divided into Arm 1, in which both HR-HPV assays were performed, and Arms 2 and 3, in which the PCR-RDB HPV or Cervista^®^ HR-HPV assay, respectively, was performed. Outcome indicators included the cases, sensitivity, negative predictive value (NPV), colposcopy referral rate and cost of identifying cervical intraepithelial neoplasia of grade 2/3 or worse (CIN2+/CIN3+).

**Results:**

Among the eligible participants, 18.4% were PCR-RDB HR-HPV-positive, while 16.9% were Cervista^®^ HR-HPV-positive, which reflects good agreement (k = 0.73). PCR-RDB cotesting identified more CIN3+ cases than Cervista^®^ cotesting in the first round of screening in Arm 1 (37 vs 32) and Arms 2/3 (252 vs 165). The sensitivity and NPV of PCR-RDB cotesting for identifying CIN3+ in Arm 1 (sensitivity: 94.9% vs 86.5%; NPV: 99.9% vs 99.7%) and Arms 2/3 (sensitivity: 95.1% vs 80.9%; NPV: 99.9% vs 99.6%) were higher than those of Cervista^®^ cotesting, but the cost was similar.

**Conclusions:**

The PCR-RDB HR-HPV genotyping and Cervista^®^ HR-HPV assay results were consistent. PCR-RDB cotesting possesses optimal cost-effectiveness for cervical cancer screening in China, which has the highest number of cases globally but low screening coverage.

## Background

Cervical cancer is the second most common cancer among women worldwide [1], and 85% of cases occur in developing countries [2]. China has the highest number of cervical cancer patients, with 98,900 new cases and 30,500 deaths each year [3] due to inadequate screening, lack of a human papillomavirus (HPV) vaccine and increased HPV infection rates [4]. Unfortunately, the first HPV vaccine was approved in China in 2017, and less than 30% of women over 21 years have been screened for cervical cancer [5]. Although nationwide cervical cancer screening programs began in 2009, due to the large population in China, the proportion of women undergoing programmatic screening is still low [5], and the main procedure for cervical cancer screening is screening at hospitals.

Although cytologic screening has effectively reduced the incidence and mortality, the accuracy of cytological results varies widely, ranging from 55 to 94% [6]. Cervical cancers and their precursors are closely related to persistent infection of high-risk human papillomavirus (HR-HPV) [7, 8]. HPV assays are more reliable and less dependent on human expertise [9] and have been applied in cervical cancer screening and management algorithms to maximize the detection rate of high-grade cervical lesions (HSILs) or worse [10]. HPV assays are more sensitive than cytology, and a negative result can better predict a low risk of developing cervical cancer [11]. An HPV test with objective results and high sensitivity that is suitable for use not only in China but also in other developing countries is highly desirable [12]. It has been reported that the negative rate of HR-HPV in cervical cancer patients can be as high as 19.4% [13] to 23.3% [14]. Combined HR-HPV and cytology screening can improve the detection rate of HPV-negative cervical cancer women and reduce missed diagnoses; it is particularly suitable for China, which has low screening coverage. Currently, the screening method of cotesting with HPV and cytology assays is the main strategy for cervical cancer screening in China.

Convenient and accurate techniques for HR-HPV detection and genotyping are urgently needed for HPV clinical diagnoses and epidemiological studies. Currently, the US Food and Drug Administration (FDA) has approved 5 HPV assays, including Hybrid Capture 2 (HC2) (Qiagen, Gaithersburg, Netherlands), Cervista^®^ (Hologic, Boston, Massachusetts, USA), Cobas HPV (Roche Molecular Diagnostics [Roche], Pleasanton, USA), Aptima (Hologic, San Diego, USA) and BD Onclarity™ HPV (Becton, Dickinson and Company, New Jersey, USA). The Cervista^®^ HR-HPV assay [15] was applied to detect 14 HR-HPV types (HPV-16, 18, 31, 33, 35, 39, 45, 51, 52, 56, 58, 59, 66 and 68) without identification of each HR-HPV type. In 2012, the ATHENA study [16] recommended that HPV-16/18-positive women be directly referred for colposcopy regardless of the cytology results, and HR-HPV genotyping assays have been increasingly used for cervical cancer screening. The PCR-reverse dot blot (PCR-RDB) HPV genotyping assay (Yaneng Bioscience Co., Shen Zhen, China) is one of the HR-HPV genotyping assays approved by the China Food and Drug Administration (CFDA) in China [17]. This method [17] is similar to Cervista^®^ HR-HPV, which also detects 14 HR-HPV types (HPV-16, 18, 31, 33, 35, 39, 45, 51, 52, 56, 58, 59, 66 and 68).

Many studies [18] have shown the great specificity and sensitivity of Cervista^®^ HR-HPV, similar to those of the HC2 assay. However, the efficacy of the Cervista^®^ HR-HPV assay combined with a cytology assay (Cervista^®^ cotesting) for cervical cancer screening is unknown. The PCR-RDB HPV genotyping assay is commonly used for cervical cancer screening in China, but the performance of PCR-RDB HPV genotyping and cytology cotesting (PCR-RDB cotesting) for cervical cancer screening is still unclear. To date, no studies have compared the efficacy of PCR-RDB cotesting and Cervista^®^ cotesting for the identification of HSIL or worse conditions in a screening population in China.

In this study, we therefore compared the cost-effectiveness of PCR-RDB HR-HPV genotyping and Cervista^®^ HR-HPV nongenotyping assays for cervical cancer screening among 32,306 Chinese women.

## Materials and methods

### Study participants

The study was conducted in accordance with the 2008 Declaration of Helsinki and was approved by the Ethics Committee of Fujian Maternity and Child Health Hospital (No. 2012-031). All participants provided written informed consent. This cohort was a programmatic, large data-based observational cohort of women who underwent cervical cancer screening in China. All participants in this study were from the Fujian Province Cervical Lesions Screening Cohorts (FCLSCs), Fujian, China. FCLSCs are cervical cancer screening cohorts established in Fujian Province, with more than 200,000 cases used to assess the value of introducing HR-HPV testing into screening. Between July 2012 and August 2015, 32, 306 women who participated in a cervical cancer screening program in Fujian Maternity and Child Health Hospital were enrolled for testing with HR-HPV and cytology assays. The participants were required to meet the following criteria: (a) aged 21–65 years; (b) gynecological patient of the hospital; (c) no history of cervical cancer or cervical intraepithelial neoplasia (CIN); and (d) no history of cervical surgery or hysterectomy. Women were excluded based on the following criteria: (a) pregnancy or recent childbirth (within 6 weeks); (b) history of vulvar intraepithelial neoplasia or worse condition or vaginal intraepithelial neoplasia or worse condition; (c) history of other malignancies; (d) history of cervical cancer screening in the past three years; (e) serious autoimmune disease or uremia; and (f) previous vaccination with an HPV vaccine.

Because the assets needed to compare two HPV assays using the same specimens in a screening population setting were limited, the enrolled participants were divided into three arms. Arm 1 was composed of participants screened for cervical cancer using the Cervista^®^ HR-HPV assay, PCR-RDB HR-HPV genotyping assay and ThinPrep^®^ Cytologic Test (TCT) simultaneously to evaluate the consistency and effectiveness of the HR-HPV results. Arm 1 consisted of all eligible participants who met the inclusion criteria and were enrolled between July 2012 and October 2012. Arm 2 was composed of participants screened for cervical cancer using the PCR-RDB HPV genotyping assay and TCT, and Arm 3 was composed of participants screened using the Cervista^®^ HR-HPV assay and TCT. Arm 2 and Arm 3 were designed to compare the cost-performance of the two HR-HPV assays for cervical cancer screening and consisted of all eligible participants who met the inclusion criteria and were enrolled between November 2012 and August 2015. Arm 2 included patients from Gynecology Districts 1–3 of Fujian Maternity and Child Health Hospital, and Arm 3 included patients from Gynecology Districts 4–5 of Fujian Maternity and Child Health Hospital.

### Screening procedures in the Arm 1 cohort

**Fig. 1:**
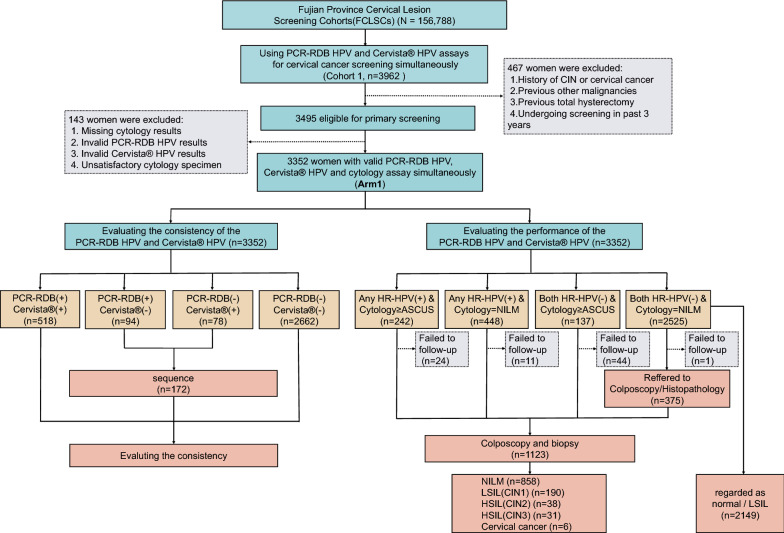
Flowchart of the screening profile in Arm 1. Arm 1, the population that underwent PCR-RDB HR-HPV genotyping, Cervista^®^ HR-HPV and cytology assays simultaneously for cervical cancer screening. HPV, human papillomavirus; PCR-RDB HPV, PCR-reverse dot blot high-risk human papillomavirus DNA genotyping assay; Cervista^®^ HPV, Cervista^®^ high-risk human papillomavirus DNA assay; PCR-RDB(−), PCR-RDB HPV assay all HR-HPV types negative; PCR-RDB(+), PCR-RDB HPV assay any of 14 h-HPV types positive; Cervista^®^ (−), Cervista^®^ HR-HPV assay all groups negative; Cervista^®^ (+), Cervista^®^ HR-HPV assay any of the groups positive; CIN, cervical intraepithelial neoplasia; NILM, negative for intraepithelial lesion or malignancy; ≥ ASCUS, atypical squamous cells of undetermined significance or worse, including atypical squamous cells without excluding high-grade lesions and atypical glandular cells; LSIL, low-grade squamous intraepithelial lesion, up to CIN1; HSIL, high-grade squamous intraepithelial lesion, including CIN2 and CIN3; Any HR-HPV, PCR-RDB HR-HPV or Cervista^®^ HR-HPV; Both HR-HPV, PCR-RDB HR-HPV and Cervista^®^ HR-HPV

Experienced gynecologists performed all gynecological examinations of the vulva, vagina and cervix for all eligible participants and performed speculum exams, collected specimens of cervical exfoliated cells with a broom brush, and transferred the cells to PreservCyt liquid (Hologic Inc., Boston, USA), which was stored at 4 ℃ until use in the cytology and HR-HPV DNA assays. According to the guidelines [16], Arm 1 included women who simultaneously underwent testing with the Cervista^®^ HR-HPV assay, PCR-RDB HR-HPV genotyping assay and TCT assay for primary cervical cancer screening. The Cervista^®^ HR-HPV assay and PCR-RDB HR-HPV genotyping assay were both performed in the same laboratory using the same cervical specimen. The cytology assay was performed in another pathology department; the laboratory staff responsible for each assay were unaware of the results of the other assay(s) when they performed the analyses. In the first round of screening, women with negative HR-HPV and cytology results were instructed to undergo routine screening after 3 years. Individuals whose samples were positive for HR-HPV types other than 16 and 18 (non-16/18) and those who had normal cytology or those with a negative HR-HPV result and cytology demonstrating atypical squamous cells of undetermined significance (ASCUS) underwent repeat HR-HPV genotyping/HR-HPV and cytology assays after 1 year. Women were referred for colposcopy and biopsy if they tested positive for HR-HPV types 16/18 regardless of the cytology results, if they tested positive for any types of HR-HPV and had cytology demonstrating ASCUS, or if the cytology results were indicative of low-grade squamous intraepithelial lesions or worse (≥ LSIL) regardless of the HR-HPV results. Colposcopy was performed by a skilled colposcopy physician. The squamous-columnar junction of the cervix was completely visible by colposcopy, which indicated that the colposcopy was satisfactory. When the colposcopy was normal, 4 sites in the cervix were randomly selected for biopsy. Conversely, when the colposcopy was abnormal, the lesioned tissue was obtained for cervical biopsy. If the colposcopy was unsatisfactory (the squamous-columnar junction was not completely visible), endocervical curettage (ECC) was performed immediately. Women with histologically confirmed cervical intraepithelial neoplasia of grade 2 or worse (CIN2+) were advised to undergo treatment according to the American Society for Colposcopy and Cervical Pathology (ASCCP) guidelines [16, 19]. All untreated women were followed up, and a second round of screening was performed 3 years later. A detailed flowchart of the screening procedure is shown in Figs. 1 and 3a, b

### Screening procedures in the Arm 2 cohort (PCR-RDB cotesting)

**Fig. 2:**
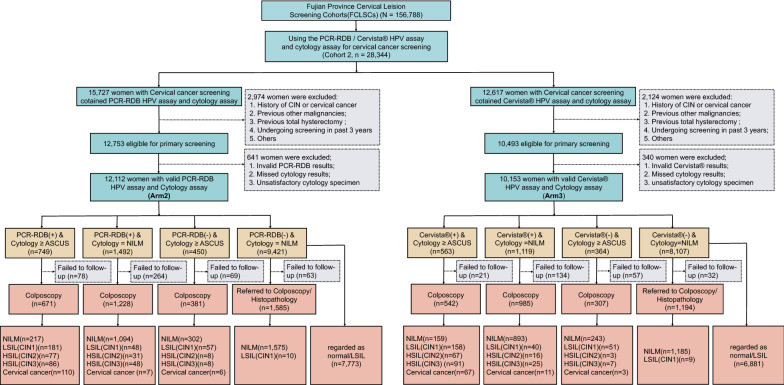
Flowchart of the screening profile in Arms 2 and 3. Arm 2, all participants screened for cervical cancer using the PCR-RDB HR-HPV genotyping assay combined with cytology assay; Arm 3, all participants screened for cervical cancer using Cervista^®^ HR-HPV assay combined with cytology assay; HPV, human papillomavirus; PCR-RDB HPV, PCR-reverse dot blot human papillomavirus DNA genotyping assay; Cervista^®^ HPV, Cervista^®^ high-risk human papillomavirus DNA assay; PCR-RDB(−), PCR-RDB HPV assay all HR-HPV types negative; PCR-RDB(+), PCR-RDB HPV assay any of 14 h-HPV types positive; Cervista^®^ (−), Cervista^®^ HR-HPV assay all groups negative; Cervista^®^ (+), Cervista^®^ HR-HPV assay any of the groups positive; CIN, cervical intraepithelial neoplasia; NILM, negative for intraepithelial lesion or malignancy; ≥ ASCUS, atypical squamous cells of undetermined significance or worse, including atypical squamous cells without excluding high-grade lesions and atypical glandular cells; LSIL, low-grade squamous intraepithelial lesion or CIN1; HSIL, high-grade squamous intraepithelial lesion, including CIN2 and CIN3

**Fig. 3:**
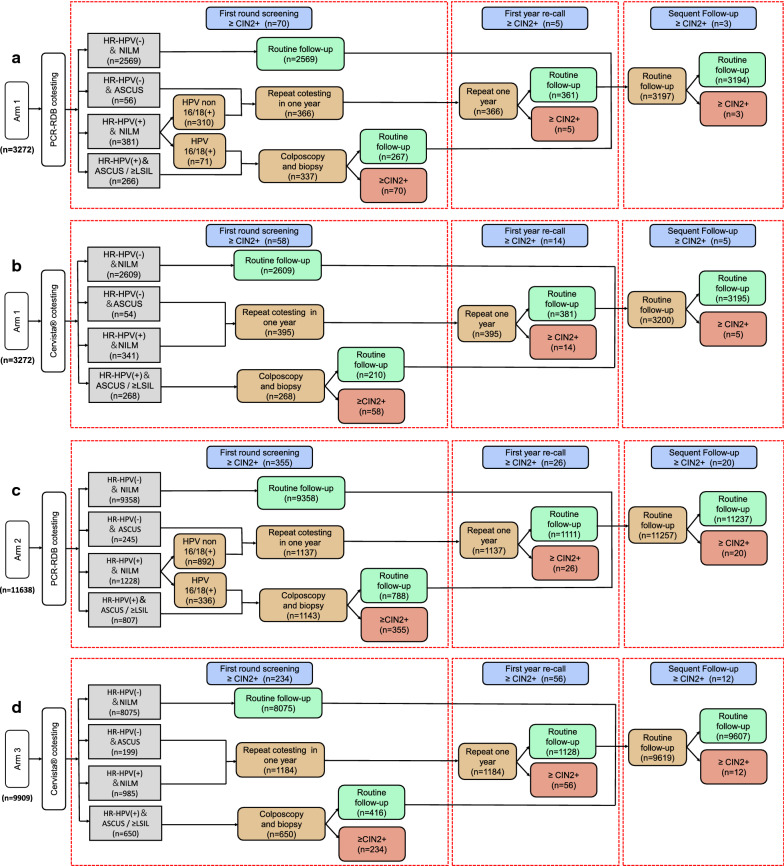
Screening procedures for Cervista^®^ cotesting and PCR-RDB cotesting in Arm 1 and Arm 2/Arm 3. **a** Cervista^®^ cotesting in Arm 1 primarily screens women with both cytology and Cervista^®^ HR-HPV assays in Arm 1 and then refers those with cytology ASCUS and HR-HPV positive/cytology LSIL or worse for colposcopy; **b** PCR-RDB cotesting with 16/18 genotyping in Arm 1 primarily screens women with both cytology and PCR-RDB HPV genotyping assays in Arm 1 and then refers those with cytology ASC-US and PCR-RDB HR-HPV positive/PCR-RDB HPV16 or HPV18 positive/cytology LSIL or worse for colposcopy; **c** Cervista^®^ cotesting in Arm 2/Arm 3, primarily screens women with both cytology and Cervista^®^ HR-HPV assays in Arm 2/Arm 3 and then refers those with cytology ASCUS and HR-HPV positive/cytology LSIL or worse for colposcopy; **d** PCR-RDB cotesting with 16/18 genotyping in Arm 2/Arm 3, primarily screens women with both cytology and PCR-RDB HPV genotyping assays in Arm 2/Arm 3 and then refers those with cytology ASCUS and PCR-RDB HR-HPV positive/PCR-RDB HPV16 or HPV18 positive/cytology LSIL or worse for colposcopy. HR-HPV, high-risk human papillomavirus; NILM, negative for intraepithelial lesion or malignancy; ASCUS, atypical squamous cells of undetermined significance; ≥ LSIL, low-grade squamous intraepithelial lesion or worse, including atypical squamous cells without excluding high-grade lesions and atypical glandular cells

For PCR-RDB HR-HPV genotyping and cytology cotesting for primary cervical screening in Arm 2 women, the two cervical exfoliated cell samples were simultaneously collected from all Arm 2 women for PCR-RDB HR-HPV genotyping and cytology assays. Cervical exfoliated cells were collected and preserved in the same manner as that in the Arm 1 women. Women with negative HR-HPV genotyping and cytology results were instructed to undergo a second routine screening after 3 years. Individuals whose samples were positive for HR-HPV non-16/18 and who had normal cytology or those with a negative HR-HPV result and cytology demonstrating ASCUS underwent repeat HR-HPV genotyping and cytology assays after 1 year. Women were referred for colposcopy and/or biopsy within 10 weeks of the collection of exfoliated cells if they tested positive for HR-HPV types 16/18 regardless of the cytology results, if they tested positive for any types of HR-HPV and had cytology demonstrating ASCUS, or if the cytology results were indicative of ≥ LSIL regardless of the HR-HPV results. Women with histologically confirmed CIN2+ were treated according to the ASCCP guidelines [16, 19]. All untreated women were followed up, and a second round of screening was performed 3 years later. A detailed flowchart of the screening procedure is shown in Figs. 2 and 3c.

### Screening procedures in the Arm 3 cohort (Cervista^®^ cotesting)

Cervical exfoliated cells were collected and preserved in the same manner as in Arm 1 women. Arm 3 included women who were simultaneously tested by the Cervista^®^ HR-HPV nongenotyping assay and cervical cytology assay for primary cervical cancer screening. In the first round of screening, women with negative HR-HPV and cytology results were routinely screened 3 years later. Women with a positive HR-HPV result and normal cytology or a negative HR-HPV result and cytology demonstrating ASCUS underwent repeat testing with HR-HPV and cytology assays after 1 year. If both the HR-HPV and cytology results were negative at the 1-year follow-up, a second round of screening was performed 3 years later; however, if the HR-HPV result was positive or cytological abnormalities were noted, the patient was immediately referred for colposcopy and/or biopsy. Women with a positive HR-HPV result and cytology demonstrating ASCUS and those with cytology results classified as ≥ LSIL regardless of the HR-HPV result immediately underwent colposcopy and/or biopsy. Subsequent management of women with histologically confirmed CIN2+ was similar to that in Arm 1. A detailed flowchart of the screening procedure is shown in Figs. 2 and 3d.

### Outcomes

The study compared key program indicators in terms of consistency, effectiveness and cost. When assessing consistency (defined as the degree of agreement between two similar assays applied to the same specimen), our goal was to evaluate whether the Cervista^®^ HR-HPV assay and PCR-RDB HR-HPV genotyping assay can identify HR-HPV infection in the same cervical exfoliated cells. We defined consistency according to the percentage and kappa value of similar results for HR-HPV detection using the same specimen, which was detected by the PCR-RDB HPV genotyping assay and the Cervista^®^ HR-HPV assay. When evaluating effectiveness, the purpose of this study was to compare the effectiveness of the PCR-RDB cotesting (PCR-RDB HPV genotyping assay) and the Cervista^®^ cotesting (Cervista^®^ HR-HPV assay) strategies in identifying CIN2+/CIN3+ lesions. The outcome was reflected in the proportion of women with pathologically identified CIN2+/CIN3+ among all screened women in the first round, 1-year follow-up and second round of screening in Arm 1 and Arm 2/Arm 3, as well as the sensitivity, specificity, positive predictive value (PPV), negative predictive value (NPV), positive likelihood ratio (PLR) and negative likelihood ratio (NLR) of the screening strategy. Sensitivity was defined as the percentage of women identified as positive by the PCR-RDB cotesting (or Cervista^®^ cotesting) among women with a cervical histopathological diagnosis of CIN2+/CIN3+. Specificity was defined as the percentage of women identified as negative by PCR-RDB cotesting (or Cervista^®^ cotesting) among women with a cervical histopathological diagnosis of normal/CIN1. The PPV was defined as the percentage of women who had a CIN2+/CIN3+ outcome (identified by cervical histopathology) out of all women who tested "positive" on PCR-RDB cotesting (or Cervista^®^ cotesting). The NPV was defined as the percentage of women who had a normal/CIN1 outcome out of all women who tested "negative" on PCR-RDB cotesting (or Cervista^®^ cotesting). Cost was defined as the cost of performing cervical cancer screening (per 1000 screened women) [20]. The following indicators were calculated at each period: (1) the cost per 1000 screened women at the first-round screening; (2) the cost per 1000 screened women at the 1-year follow-up; (3) total cost per 1000 screened women per round of screening; and (4) the cost per identified CIN2+ woman per round of screening. For the cost calculations in this study, we considered only direct medical expenses and excluded indirect nonmedical expenses. Direct medical expenses were defined as costs (in dollars) that women must pay to the hospital for cervical cancer screening. Unit costs were taken from the Fujian medical price database.

### Cervista^®^ HR-HPV assay

The Cervista^®^ (Hologic Inc., Madison, WI, USA) HR-HPV assay is a qualitative test used to detect 14 HR-HPV types (16, 18, 31, 33, 35, 39, 45, 51, 52, 56, 58, 59, 66, and 68). The Cervista^®^ HR-HPV assay uses the Invader™ chemistry, a signal amplification method for detection of specific nucleic acid sequences of HR-HPV. The assay uses three separate oligonucleotide mixtures: Mix 1 (Species A5/A6) contains HPV-51, -56 and -66; Mix 2 (Species A7) contains HPV-18, -39, -45, -59 and -68; and Mix 3 (Species A9) contains HPV-16, -31, -33, -35, -52 and -58. The detailed experimental procedures of the Cervista^®^ HR-HPV assay can be found online (https://www.hologic.com/sites/default/files/package-insert/15-3100_105_01.pdf). Our study and previous studies [21, 22] have demonstrated that the Cervista^®^ HR-HPV assay is an effective HR-HPV detection method.

### PCR-RDB HR-HPV genotyping assay

The PCR-RDB HPV genotyping assay (Yaneng Bioscience Co., Ltd., China) can detect 14 HR-HPV genotypes (HPV-16, -18, -31, -33, -35, -39, -45, -51, -52, -56, -58, -59, -66, -68), and the type distribution was the same as that of the Cervista^®^ HR-HPV assay. The PCR-RDB HR-HPV genotyping assay is based on the PCR and reverse dot hybridization method for detecting the 14 HR-HPV genotypes. The PCR-RDB HPV genotyping assay was performed according to the manufacturer's instructions. Briefly, an aliquot of a 5-μL DNA sample was used. HPV was amplified in a thermal cycler under the following conditions: 50 °C for 15 min and 95 °C for 10 min, followed by a total of 40 cycles of 94 °C for 10 s, 45 °C for 90 s, and 72 °C for 30 s. The PCR products were immobilized onto a nitrocellulose membrane and hybridized with typing probes. After amplification, HPV genotyping was performed by RDB hybridization on nitrocellulose membrane strips fixed with different HPV type-specific probes. Our study and previous studies [17, 23] have demonstrated that the PCR-RDB HR-HPV genotyping assay is an effective HR-HPV genotyping detection method.

### Liquid-based cytology

All liquid-based cytology specimens were blinded and independently evaluated by 2 experienced cytopathologists. If the diagnoses differed, the sample was reviewed again, and a consensus diagnosis was obtained. The results were analyzed using the Bethesda system. Samples were classified as negative for intraepithelial lesion or malignancy (NILM), ASCUS, LSIL, atypical squamous cells, and it was not possible to exclude high-grade squamous intraepithelial lesions (ASC-H), HSIL, squamous cervical cancer (SCC) and atypical glandular cells (AGC).

### Histology

Women with a punch biopsy diagnosis of HSIL or higher were treated with cold knife conization or a loop electrosurgical excision procedure (LEEP). Formalin (10%) was used to fix specimens, which were routinely processed for paraffin embedding. Subsequently, nearly 4-μm-thick histological sections were cut and stained with hematoxylin and eosin according to the standard instructions. Then, cervical biopsy specimens were histologically examined and classified according to the CIN system.

### Sequencing

Products of the HPV L1 gene amplified from samples by nested PCR using type-specific primers were purified using the QIAquick PCR Purification Kit (Qiagen, Hilden, Germany) according to the manufacturer’s instructions and sequenced by Sangon Biotech Co., Ltd. (Shanghai, China). The primer sequences for PCR amplification of HPV L1 were GP5+ (5′-TTTGTTACTGTGGTAGATACTAC-3′) and GP6+ (5′-GAAAAATAAACTGTAAATCATATTC-3′). The nested PCR amplification conditions were as follows: 50 °C for 15 min and 95 °C for 10 min; 10 cycles for 10 s at 94 °C, 90 s at 42 °C, and 30 s at 72 °C; 30 cycles for 10 s at 94 °C, 60 s at 46 °C, and 20 s at 72 °C; and a final extension for 5 min at 72 °C. The resulting DNA sequences were compared with the sequences of known HPV types using the basic local alignment search tool from the NCBI website (https://www.ncbi.nlm.nih.gov/BLAST).

### Statistical analysis

The consistency of the PCR-RDB HPV genotyping and Cervista^®^ HR-HPV assays was assessed using Cohen's kappa value. Kappa (k) values of 0–0.2, 0.21–0.4, 0.41–0.6, 0.61–0.8, 0.81–0.99, and 1.0 represented poor, slight, medium, good, almost perfect, and perfect consistency, respectively. The performance of the PCR-RDB HPV genotyping assay and the Cervista^®^ HR-HPV assay was assessed using the sensitivity, specificity, NPV, PPV, NLR, and PLR. For the screening strategies, the sensitivity, specificity, NPV, PPV, cost per 1000 screened women at first-round screening, cost per 1000 screened women at the 1-year follow-up, total cost per 1000 screened women per round of screening, cost per identified CIN2+ woman per round of screening, and number of colposcopies needed to detect one case of CIN2+ or CIN3+ were evaluated. Analyses were performed using SPSS 22.0 (IBM, New York, USA) and MedCalc 18.11.3 software (MedCalc, Ostend, Belgium). A two-tailed *P*-value < 0.05 was considered statistically significant.

## Results

### Comparison of the consistency of the PCR-RDB HPV genotyping and Cervista^®^ HR-HPV assays for detecting HR-HPV infection

In total, 32,306 women who participated in the cervical cancer screening program were enrolled for testing with HR-HPV and cytology assays in this study. Overall, 5565 women were excluded because they met the exclusion criteria, and 1124 women were excluded because of inadequate cervical exfoliated cell specimens, invalid HR-HPV results, or invalid cytology results. Finally, 25,617 women with valid HR-HPV and cytological tests (Arm 1 = 3352, Arm 2 = 12,112, Arm 3 = 10,153) were included. The average age of valid participants was 36.8 ± 10.1 years (range 21 to 65 years), and the average ages of the participants in Arm 1, Arm 2, and Arm 3 were 37.4 ± 10.1, 37.3 ± 10.0 and 36.3 ± 9.7 years, respectively. The mean ages of first sexual intercourse in Arm 1, Arm 2, and Arm 3 were 16.87 ± 5.43, 16.99 ± 5.81 and 17.01 ± 4.89 years, respectively (*p* = 0.268). There were no differences in the time of pregnancy, smoking background, drinking background, and degree of education among the women in Arm 1, Arm 2, and Arm 3 (*p* = 0.183, 0.669, 0.744, 0.615, respectively; Additional file 1: Table S1). A total of 15,464 participants had valid PCR-RDB HPV results in Arm 1 (n = 3352) and Arm 2 (n = 12,112), and 18.4% (2853/15,464) of participants were PCR-RDB HR-HPV-positive, including 5.2% (813/15,464) who were PCR-RDB HPV-16/18-positive (HPV-16 and/or HPV-18 positive) and 13.2% (2040/15,464) who were PCR-RDB non-HPV-16/18-positive (both HPV-16 and HPV-18 were negative, but any one or more types of HPV-31/-33/-35/-39/-45/-51/-52/-56/-58/-59/-66/-68 were positive). A total of 13,505 participants had valid Cervista^®^ HR-HPV results in Arm 1 (n = 3352) and Arm 3 (n = 10,153); 16.9% (2278/13,505) of samples were Cervista^®^ HR-HPV-positive, including 11.4% (1534/13,505) in the Cervista^®^ A9 group and 5.5% (744/13,505) in the Cervista^®^ non-A9-positive group (Cervista^®^ HR-HPV A9 group negative, but A5/A6 group and/or A7 group positive). The HR-HPV positivity rate of the PCR-RDB genotyping assay showed two age peaks, 21–24 (20.1%) and 50–65 (24.0%) years, but PCR-RDB HPV-16/18 positivity rates did not show an age association. Similar results were obtained for the Cervista^®^ HR-HPV assay, as shown in Additional file 2: Figure S1A. Detailed results for the two HPV assays in different age subgroups, cytology subgroups and pathology subgroups are shown in Additional file 2: Figure S1. Of the 25,617 participants, 2.9% (748/25,617) of the women were diagnosed with CIN2+ and 2.0% were diagnosed (501/25,617) with CIN3+ during the first-round screening and 1-year follow-up.

**Fig. 4:**
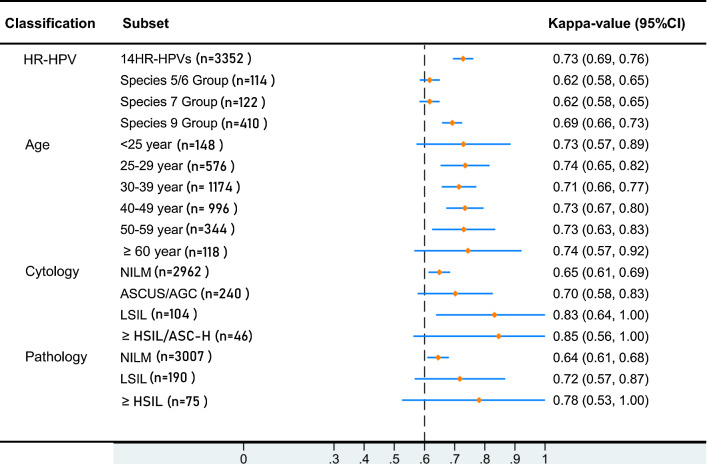
Consistency of the PCR-RDB HR-HPV genotyping assay and Cervista^®^ HR-HPV assay in different age/cytology/pathology groups. CI, Confidence interval; 14HR-HPVs, 14 high-risk human papillomavirus types, containing HPV-16, -18, -31, -33, -35, -39, -45, -51, -52, -56, -58, -59, -66, and -68; Species 5/6 group, high-risk human papillomavirus species 5 and species 6, containing HPV-51, 56 and -66; Species 7 group, high-risk human papillomavirus species 7, containing HPV-18, -39, -45, -59 and -68; Species 9 group, high-risk human papillomavirus species 9, containing HPV-16, -31, -33, -35, -52 and -58; NILM, negative for intraepithelial lesion or malignancy; ASCUS/AGC, atypical squamous cells of undetermined significance/atypical glandular cells; LSIL, low-grade squamous intraepithelial lesion, i.e., CIN1; ≥ HSIL/ASC-H, high-grade squamous intraepithelial lesion or worse/atypical squamous cells without excluding high-grade squamous intraepithelial lesion;  , Kappa value;  , 95% confidence interval for the kappa value

Consistency analysis indicated that the results of the two HPV assays showed good agreement (k = 0.73, 95% confidence interval [CI] 0.69–0.76). This consistency remained for women in different age groups. However, the consistency of the two HPV assays for LSIL (k = 0.83, 95% CI 0.64–1.00)/HSIL (k = 0.85, 95% CI 0.56–1.00) cytology samples was higher than that in the cytology-negative samples (k = 0.65, 95% CI 0.61–0.69). Detailed results for the consistency analysis are shown in Fig. 4. In total, 94 samples were PCR-RDB HR-HPV-positive but Cervista^®^ HR-HPV-negative, and 78 samples were PCR-RDB HR-HPV-negative but Cervista^®^ HR-HPV-positive. Sixty samples were PCR-RDB HPV-16/31/33/35/52/58 (A9 group)-positive but Cervista^®^ HR-HPV A9 group-negative, and 42 samples were PCR-RDB HPVA9 group-negative but Cervista^®^ HR-HPV A9 group-positive (Additional file 3: Table S2).

### Cost-effectiveness of PCR-RDB HPV genotyping cotesting (PCR-RDB cotesting) and Cervista^®^ HR-HPV cotesting (Cervista^®^ cotesting) for identifying CIN2+ and CIN3+ lesions

Nearly 798 participants were lost to recall during the follow-up period. Finally, 24,819 women (Arm 1 = 3272, Arm 2 = 11,638, Arm 3 = 9909) had eligible follow-up results and valid pathology results and were included in the cost-performance analysis. As shown in Tables 1 and 2, in Arm 1, the sensitivity of the PCR-RDB cotesting strategy (93.3%) for identifying CIN2+ was significantly higher than that of the Cervista^®^ cotesting strategy (80.6%) in the first round of screening. Similar results were observed for CIN3+. The sensitivity, specificity, PPV, and NPV of the Cervista^®^ cotesting strategy for identifying CIN2+ in Arm 2/Arm 3 in the first round of screening were 80.7%, 95.7%, 36.0%, and 99.4%, respectively, showing good effectiveness. PCR-RDB cotesting in Arm 2/Arm 3 showed a higher sensitivity (93.2% vs 80.7%) and NPV (99.8% vs 99.4%) but a slightly lower specificity (93.0% vs 95.7%) and PPV (31.1% vs 36.0%) than the Cervista^®^ cotesting strategy; moreover, the PCR-RDB required more colposcopies (3.22 vs 2.78) to identify one case of CIN2+. In terms of economic cost, the total cost of a round of cervical cancer screening using PCR-RDB cotesting for 1000 women was similar to that using Cervista^®^ cotesting [PCR-RDB cotesting vs Cervista^®^ cotesting = $57,932 vs $57,922 (in Arm 1) or $56,993 vs $56,902 (in Arms 2/3)]. However, the PCR-RDB cotesting screening strategy cost less than the Cervista^®^ cotesting strategy in terms of the cost of identifying each CIN2+ woman [PCR-RDB cotesting vs Cervista^®^ cotesting = $2527 vs $2632 (in Arm 1) or $1654 vs $1867 (in Arms 2/3)]. Similar results were observed for CIN3+.

**Table 1:** The performances of PCR-RDB genotyping cotesting and Cervista^®^ cotesting for identifying CIN2+. (N = 24,819)

	PCR-RDB cotesting	Cervista^®^ cotesting	*P*-value
Arm 1 (n = 3272)			
Sensitivity	93.3% (87.7–98.9%)	80.6% (71.4–89.7%)	0.019
Specificity	91.6% (90.7–92.6%)	93.4% (92.6–94.3%)	0.007
PPV	20.8% (18.8–23.2%)	21.6% (19.9–23.9%)	0.873
NPV	99.8% (99.8%–100.0%)	99.5% (99.3–99.7%)	0.031
PLR	11.4 (9.8–12.7)	12.6 (10.3–14.6)	/
NLR	0.07 (0.03–0.17)	0.21 (0.13–0.33)	/
No. of cases identified at first round screening	70	58	/
No. of cases identified at 1-year follow-up	5	14	/
No. of cases identified at sequent follow-up	3	5	/
No. of colposcopies to identified 1 case at first round	4.81	4.62	/
Cost per 1000 screened women at first round screening^a^	$ 52,292 (¥ 366,044)	$ 51,238 (¥ 358,666)	/
Cost per 1000 screened women at 1-year follow-up	$ 5640 (¥ 39,480)	$ 6684 (¥ 46,788)	/
Total cost per 1000 screened women per round	$ 57,932 (¥ 405,524)	$ 57,922 (¥ 405,454)	/
Cost per each identified CIN2+ women per round	$ 2527 (¥ 17,689)	$ 2632 (¥ 18,424)	/
Arm 2 (n = 11,638) vs. Arm 3 (n = 9909)^b^			
Sensitivity	93.2% (90.6–95.7)	80.7% (76.2–85.2)	< 0.001
Specificity	93.0% (92.5–93.5)	95.7% (95.3–96.1)	< 0.001
PPV	31.1% (28.4–33.7)	36.0% (32.3–39.7)	0.037
NPV	99.8% (99.7–99.9)	99.4% (99.2–99.6)	< 0.001
PLR	13.8 (12.4–14.3)	19.2 (16.7–20.8)	/
NLR	0.07 (0.05–0.11)	0.20 (0.16–0.26)	/
No. of cases identified at first round screening	355	234	/
No. of cases identified at 1-year follow-up	26	56	/
No. of cases identified at sequent follow-up	20	12	/
No. of colposcopies to identified 1 case at first round screening	3.22	2.78	/
Cost per 1000 screened women at first round screening	$ 52,053 (¥ 364,371)	$ 50,422 (¥ 352,954)	/
Cost per 1000 screened women at 1-year follow-up	$ 4940 (¥ 34,580)	$ 6480 (¥ 45,360)	/
Total cost per 1000 screened women per round	$ 56,993 (¥ 398,951)	$ 56,902 (¥ 398,314)	/
Cost per each identified CIN2+ women per round	$ 1654 (¥ 11,578)	$ 1867 (¥ 13,069)	/

PCR-RDB cotesting, primarily screens women with both cytology and PCR-RDB HR-HPV genotyping assays, and then refers those with cytology ASCUS and PCR-RDB HR-HPV positive/PCR-RDB HPV16 or HPV18 positive/cytology LSIL or worse to colposcopy; Cervista^®^ cotesting, primarily screens women with both cytology and Cervista^®^ HR-HPV assays, and then refers those with cytology ASC-US and HR-HPV positive/cytology LSIL or worse to colposcopy; Arm 1, composed of participants screened for cervical cancer using the Cervista^®^ HR-HPV assay, PCR-RDB HPV genotyping assay and ThinPrep^®^ Cytologic Test (TCT) simultaneously; Arm 2, composed of participants screened for cervical cancer using the PCR-RDB HR-HPV genotyping assay and the TCT; Arm 3, composed of participants screened using the Cervista^®^ HR-HPV assay and the TCT

CIN2+, cervical intraepithelial neoplasia grade 2 or worse; PPV, positive predictive value; NPV, negative predictive value; PLR, positive likelihood ratio; NLR, negative likelihood ratio

^a^ Calculate only the cost of screening women for cervical cancer screening in hospitals

^b^ Compare Arm 2′s screening strategy with Arm 3′s screening strategy

**Table 2:** The performances of PCR-RDB genotyping cotesting and Cervista^®^ cotesting for identifying CIN3+. (N = 24,819)

	PCR-RDB cotesting	Cervista^®^ cotesting	*P*-value
Arm 1 (n = 3272)			
Sensitivity	94.9% (87.9–100.0%)	86.5% (75.5–97.5%)	0.020
Specificity	90.6% (89.6–91.6%)	92.7% (91.8–93.6%)	0.002
PPV	10.8% (7.9–13.0%)	11.9% (7.3–12.1%)	0.760
NPV	99.9% (99.8–100.0%)	99.7% (99.6–99.9%)	0.046
PLR	10.2 (8.8–11.5)	12.0 (9.9–14.2)	/
NLR	0.06 (0.01–0.22)	0.15 (0.06–0.33)	/
No. of cases identified at first round screening	37	32	/
No. of cases identified at follow-up round	2	5	/
No. of colposcopies to identified 1 case at first round	9.1	8.4	/
Cost per each identified CIN3+ women per round	$ 4860 (¥ 34,020)	$ 5122 (¥ 35,854)	/
Arm 2 (n = 11,638) vs. Arm 3 (n = 9909)^a^			
Sensitivity	95.1% (92.5–97.7)	80.9% (75.5–86.3)	< 0.001
Specificity	92.2% (91.7–92.7)	95.0% (94.6–95.4)	< 0.001
PPV	22.1% (19.6–24.5)	25.4% (22.0–28.7)	0.121
NPV	99.9% (99.8–99.9)	99.6% (99.5–99.7)	< 0.001
PLR	12.4 (11.3–13.0)	16.5 (14.3–18.1)	/
NLR	0.05 (0.03–0.09)	0.20 (0.15–0.27)	/
No. of cases identified at first round screening	252	165	/
No. of cases identified at follow-up round	21	43	/
No. of colposcopies to identified 1 case at first round	4.5	3.9	/
Cost per each identified CIN3+ women per round	$ 2430 (¥ 17,010)	$ 2711 (¥ 18,977)	/

CIN3+, cervical intraepithelial neoplasia grade 3 or worse; PPV, positive predictive value; NPV, negative predictive value; PLR, positive likelihood ratio; NLR, negative likelihood ratio

^a^ Compare Arm 2′s screening strategy with Arm 3′s screening strategy

## Discussion

As the largest developing country, cervical cancer poses a significant health burden in China. The PCR-RDB HPV genotyping assay is a PCR-based method used to detect and identify viral DNA from 14 HR-HPV genotypes. Some studies have confirmed that HPV-16/18 genotyping has excellent triage performance in HPV-positive women [24]. Therefore, in 2012, the ASCCP recommended that HPV-16/18-positive women be directly referred for colposcopy, regardless of their cytology results [16]. The PCR-RDB HPV genotyping assay is widely used in China because it can detect 14 HR-HPV genotypes separately. Our previous research [17] revealed that the PCR-RDB HPV genotyping assay provided a sensitive and reliable method for clinical applications of cervical cancer screening. The Chinese Society of Colposcopy & Cervical Pathology (CSCCP) guidelines recommend combined screening using HR-HPV and cytology as the primary method for cervical cancer screening in China [17, 25]. However, no studies have evaluated the efficacy of PCR-RDB cotesting in the identification of HSIL or worse conditions in China.

In this study, we used Cervista^®^ cotesting as a reference screening strategy and compared the cost-effectiveness of PCR-RDB cotesting for cervical cancer screening in China. In Arm 1 and Arms 2/3, we found that PCR-RDB cotesting had a higher sensitivity for identifying CIN2+ in the first round of screening. PCR-RDB cotesting can result in the detection of more CIN2+ women than Cervista^®^ cotesting in the first round of screening; thus, implementing the PCR-RDB cotesting screening strategy will result in earlier detection of precancerous lesions and cervical cancer. The discovery of CIN2+ lesions earlier can result in prompt intervention or treatment of patients, which can help prevent progression to severe cervical cancer and the subsequent loss of the chance for a cure [26]. Our research confirms that the implementation of the PCR-RDB cotesting screening strategy in regions with a high burden of cervical cancer will help that region recognize the WHO cervical cancer elimination targets as soon as possible [26].

The positivity rate of 14 genotypes of HR-HPV was 18.4% with the PCR-RDB HR-HPV genotyping assay, which is slightly higher than that with the Cervista^®^ HR-HPV (16.9%) and higher than that reported by Xiumin Zhao et al. [27] (9.9%). This result may have been obtained because the participants in the study were screened in hospitals rather than in the community. After age stratification, the positivity rate of the PCR-RDB HR-HPV genotyping and Cervista^®^ HR-HPV assays had two age peaks, 21–24 years and 50–65 years, which was similar to the findings from other studies from China [17, 27–29], Japan [30] and Chile [31], but HR-HPV prevalence commonly declines after the age of 25 in European and North American women [32, 33]. The main cause of the first peak of HPV infection is that women aged 21–24 are sexually active. The second peak of HR-HPV infection may be due to factors such as increased extramarital sexual behavior of the women or their husbands or decreased immunity, which could lead to infection with HPV with a low replication status [34]. In our study, good consistency was found between the PCR-RDB HR-HPV genotyping and Cervista^®^ HR-HPV assays (k = 0.73). However, the consistency among HPV test results was lowest in the cytology NILM group (k = 0.65). This finding may be explained by the very low viral load that is near the cutoff value of the HPV test, leading to poorly judged results in this subgroup [35]. Some studies [36, 37] have also confirmed this problem.

Previous studies have shown that Cervista^®^ and HC2 have similar properties in terms of HSIL identification, although their principles are different [18]. Therefore, the results of this study indicated that the efficiency of PCR-RDB cotesting was significantly higher than that of Cervista^®^ HR-HPV cotesting for predicting CIN2+/CIN3+, but the total cost of the two screening methods was similar for every 1000 women screened ($57,932 vs $57,922 in Arm 1; $56,993 vs $56,902 in Arms 2/3). However, the cost of PCR-RDB cotesting was lower than that of Cervista^®^ cotesting for each CIN2+/CIN3+ patient identified ($2527 vs $2632 in Arm 1; $1654 vs $1867 in Arms 2/3). Cost is an important factor to consider when formulating screening strategies, especially in low- and middle-income countries where resources are scarce. This study revealed that PCR-RDB cotesting may be a more cost-effective alternative to cervical cancer screening strategies in low- and middle-income countries, which have low screening coverage and large populations.

The PCR-RDB HR-HPV genotyping cotesting strategy revealed a subgroup of HPV-16/18-positive but cytologically normal women. In this study, 25.3% (407/1609) of HR-HPV-positive but cytologically normal women were HPV-16/18-positive in Arm 1 and Arm 2/3, and all of them were directly referred for colposcopy. This procedure resulted in the detection of CIN2+ lesions in an additional 19.1% (81/425) of patients during the first round of screening, confirming the importance of a PCR-RDB genotyping cotesting program for early detection of cervical lesions. Many studies have confirmed that HPV-16/18 infection has a higher carcinogenicity than any other type [38]. Therefore, HR-HPV genotyping cotesting can be used to directly refer HPV-16/18-positive women for colposcopy to achieve early diagnosis and reduce misdiagnosis of CIN2+ lesions, especially in low- and middle-income countries, which have low screening coverage.

The follow-up of cervical cancer screening is difficult in low- and middle-income countries. In this study, all women with serious abnormal screening results in the first round of screening were directly referred for colposcopy, while those with slight abnormalities will undergo repeat HR-HPV and TCT testing one year later. To increase the follow-up rate of women with abnormal screening results, we have established telephone follow-up and online follow-up for women with abnormal screening to detect the progression of cervical disease over time and reduce the rate of missed follow-up. All colposcopy and pathological examinations in this study were performed by two expert colposcopy physicians or pathologists. We also regularly provide technical training for colposcopy physicians and pathologists to improve the accuracy of colposcopy diagnosis and pathological diagnosis.

There are some limitations of this study that should be noted. First, some women with HR-HPV (non-HPV-16/18)-positive or cytology-positive results alone were not referred for colposcopy; thus, the evaluation of the specificity and PPV of the HPV assay may have been affected. Second, some women were not followed up after receiving abnormal screening results, and the loss to follow-up rate in our study was 3.1% (798/25,617), which is similar to what has been reported in other studies [39]. Although our loss to follow-up was within the allowable range, these women were at high risk of CIN2+/CIN3+, which may have led to reduced sensitivity of the study results. In future studies, telephone follow-up is needed to reduce the rate of loss of follow-up and detect disease development of patients to show more realistic results.

## Conclusions

In summary, the results of this study indicate that the PCR-RDB HPV genotyping assay and the Cervista^®^ HR-HPV assay are effective for detecting CIN2+ and CIN3+. Additionally, the strategy of PCR-RDB HPV cotesting for the 16/18 genotypes presents a higher sensitivity and NPV than the standard strategy of Cervista^®^ HR-HPV cotesting without increasing the cost. The PCR-RDB HPV genotyping assay as a cotest for the 16/18 genotypes possesses optimal cost-effectiveness for screening and should be recommended for cervical cancer screening programs in China and other developing countries.

## Supplementary information

**Additional file 1: Table S1.** Clinical characteristics of women in different screening arms. (N = 25617).

**Additional file 2: Figure S1.** Prevalence of HPV positivity in different age/cytology/pathology groups. A. Prevalence of HPV positivity in different age groups. B. Prevalence of HPV positivity in different cytology groups. C. Prevalence of HPV positivity in different pathology groups. PCR-RDB positivity, positive for any of the 14 HR-HPV types; PCR-RDB HPV-16/18 positivity, positive for either genotype 16 or 18, with or without positivity for other HPV types; Cervista positivity, positive for any of the three HR-HPV groups; Cervista A9 positivity, positive for the A9 group, with or without positivity for the two other groups; NILM, negative for intraepithelial lesion or malignancy; ASCUS, atypical squamous cells of undetermined significance; LSIL, low-grade squamous intraepithelial lesion; HSIL, high-grade squamous intraepithelial lesion; AGC, atypical glandular cells; ASC-H, atypical squamous cells without excluding high-grade squamous intraepithelial lesions; SCC, squamous cervical cancer; CIN, cervical intraepithelial neoplasia.

**Additional file 3: Table S2.** Disagreement between PCR-RDB HPV genotyping and Cervista® HR-HPV assays in different cytology subgroups (n = 172)

## Data Availability

All data generated or analyzed during this study are included in this published article and its additional information files.

## Abbreviations

HPV: Human papillomavirus
HR-HPV: High-risk human papillomavirus
Cervista^®^ cotesting: Cervista^®^ HR-HPV nongenotyping and cytology cotesting
PCR-RDB cotesting: PCR-reverse dot blot HR-HPV genotyping and cytology cotesting
LSIL: Low-grade squamous intraepithelial lesions
HSILs: High-grade cervical lesions
CIN: Cervical intraepithelial neoplasia
ASCUS: Atypical squamous cells of undetermined significance
FCLSCs: Fujian Province Cervical Lesions Screening Cohorts
LEEP: Loop electrosurgical excision procedure
