# Supplementary material for: Cost-effectiveness and accuracy of cervical cancer screening with a high-risk HPV genotyping assay vs a nongenotyping assay in China: an observational cohort study
Source: Cancer Cell Int. 2020 Aug 28;20:421. doi: 10.1186/s12935-020-01512-4 (PMC7453699; doi:10.1186/s12935-020-01512-4)
Supplement: Supplementary file 1 — Additional file 1: Table S1. Clinical characteristics of women in different screening arms. (N = 25617). [file 12935_2020_1512_MOESM1_ESM.doc]

**Table S1. Clinical characteristics of women in different screening arms. (N=25617**)

| **Variables** | **Arm1**  **(n=3352)** | **Arm2**  **(n=12112)** | **Arm3**  **(n=10153)** | ***P*-value** |
| --- | --- | --- | --- | --- |
| Age, mean ± SD | 37.4 ± 10.1 | 37.3 ± 10.0 | 36.3 ± 9.7 | 0.082 |
| Age of first sexual intercourse, mean ± SD | 16.87±5.43 | 16.99±5.81 | 17.01±4.89 | 0.268 |
| Age of marriage, mean ± SD | 26.41±3.22 | 26.11±2.85 | 26.24±2.66 | 0.342 |
| Pregnancy Times, %(n) |  |  |  |  |
| ≤2 | 59.9% (2008) | 58.2% (7054) | 59.0% (5990) | 0.183 |
| >2 | 40.1% (1344) | 41.8% (5058) | 41.0% (4163) |
| Smoking history, %(n) |  |  |  |  |
| Yes-At least once a week | 1.6% (54) | 1.4% (170) | 1.5% (149) | 0.669 |
| No | 98.4% (3298) | 98.6% (11942) | 98.5% (10004) |
| Drinking history, %(n) |  |  |  |  |
| Yes--At least once a week | 12.3% (413) | 12.8% (1550） | 12.8% (1299) | 0.744 |
| No | 87.7% (2939) | 87.2% (10562) | 87.2% (8854) |
| Degree of education, %(n) |  |  |  |  |
| <Higher education | 68.5% (2296) | 69.1% (8371) | 69.4% (7046) | 0.615 |
| ≥Higher education | 31.5% (1056) | 30.9% (3741) | 30.6% (3107) |
| HR-HPV, %(n) |  |  |  |  |
| Negative | 81.7% (2740) | 81.5% (9871) | 83.6% (8487) | <0.001 |
| Positive | 18.3% (612) | 18.5% (2241) | 16.4% (1666) |
| Cytology, %(n) |  |  |  |  |
| Normal | 90.5% (3034) | 90.1% (10912) | 90.7% (9224) | 0.158 |
| Abnormal | 9.5% (318) | 9.9% (1200) | 9.3% (929) |

**Notes:** Arm 1, composed of participants screened for cervical cancer using the Cervista® HR-HPV assay, PCR-RDB HPV genotyping assay and ThinPrep® Cytologic Test (TCT) simultaneously; Arm 2, composed of participants screened for cervical cancer using the PCR-RDB HR-HPV genotyping assay and the TCT; Arm 3, composed of participants screened using the Cervista® HR-HPV assay and the TCT.

**Abbreviations:** SD, standard deviation; HR-HPV, high-risk human papillomavirus, including HPV16, 18, 31, 33, 35, 39, 45, 55, 52, 52, 56, 59, 66, 68 genotypes.
