# Supplementary figures and images for: Cost-effectiveness and accuracy of cervical cancer screening with a high-risk HPV genotyping assay vs a nongenotyping assay in China: an observational cohort study
Source: Cancer Cell Int. 2020 Aug 28;20:421. doi: 10.1186/s12935-020-01512-4 (PMC7453699; doi:10.1186/s12935-020-01512-4)

**A**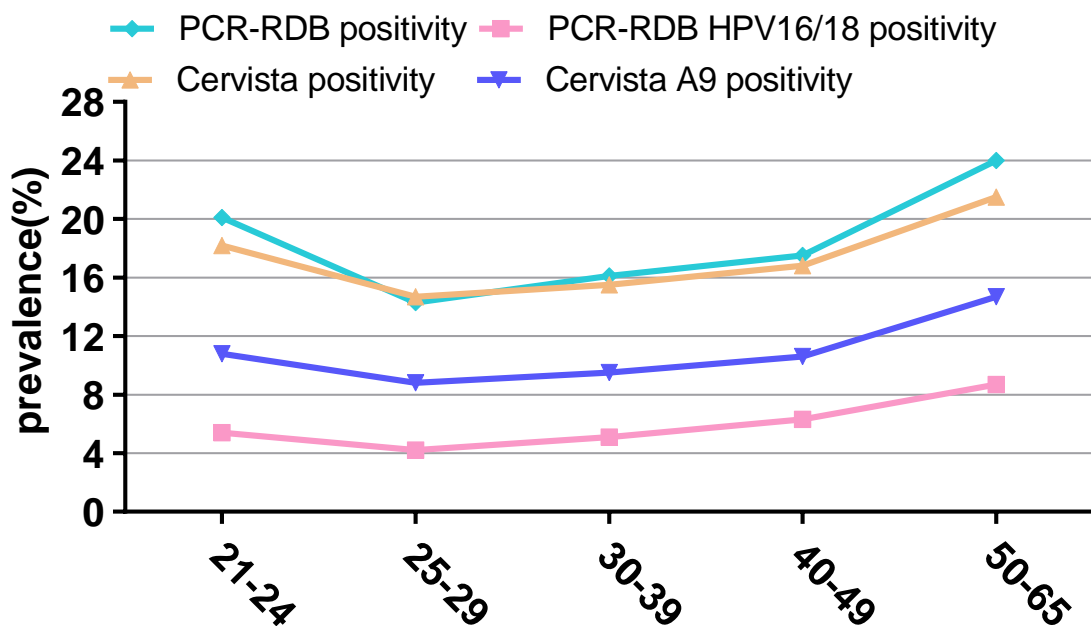**B**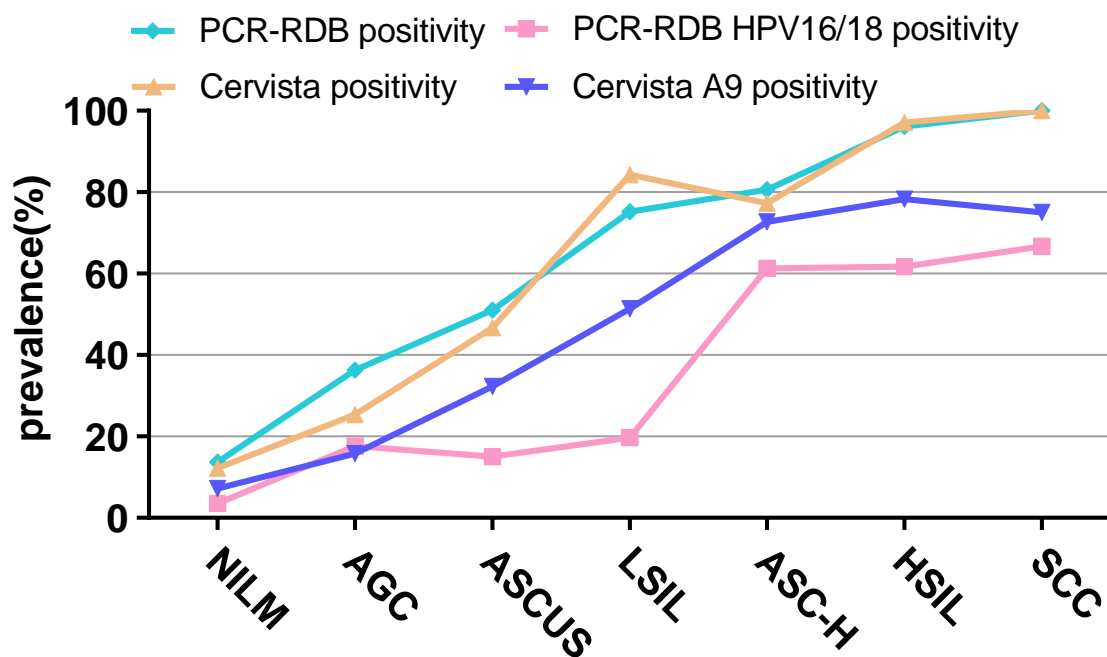**C**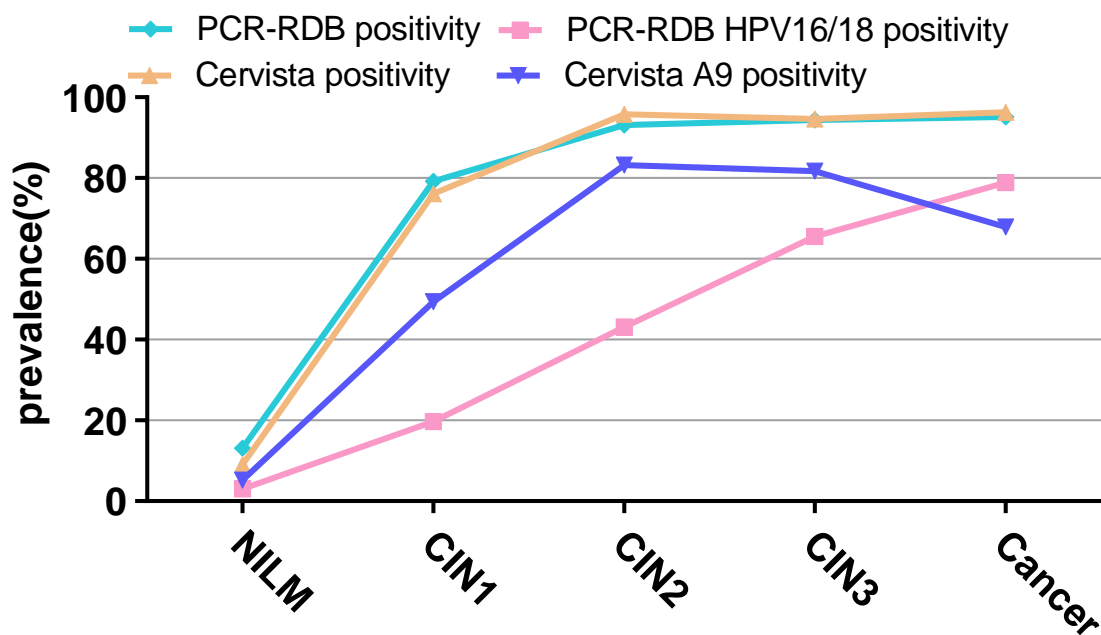

Supplement: Supplementary file 2 — Additional file 2: Figure S1. Prevalence of HPV positivity in different age/cytology/pathology groups. A. Prevalence of HPV positivity in different age groups. B. Prevalence of HPV positivity in different cytology groups. C. Prevalence of HPV positivity in different pathology groups. PCR-RDB positivity, positive for any of the 14 HR-HPV types; PCR-RDB HPV-16/18 positivity, positive for either genotype 16 or 18, with or without positivity for other HPV types; Cervista positivity, positive for any of the three HR-HPV groups; Cervista A9 positivity, positive for the A9 group, with or without positivity for the two other groups; NILM, negative for intraepithelial lesion or malignancy; ASCUS, atypical squamous cells of undetermined significance; LSIL, low-grade squamous intraepithelial lesion; HSIL, high-grade squamous intraepithelial lesion; AGC, atypical glandular cells; ASC-H, atypical squamous cells without excluding high-grade squamous intraepithelial lesions; SCC, squamous cervical cancer; CIN, cervical intraepithelial neoplasia. [file 12935_2020_1512_MOESM2_ESM.pdf]
