# Supplementary material for: Cost-effectiveness and accuracy of cervical cancer screening with a high-risk HPV genotyping assay vs a nongenotyping assay in China: an observational cohort study
Source: Cancer Cell Int. 2020 Aug 28;20:421. doi: 10.1186/s12935-020-01512-4 (PMC7453699; doi:10.1186/s12935-020-01512-4)
Supplement: Supplementary file 3 — Additional file 3: Table S2. Disagreement between PCR-RDB HPV genotyping and Cervista® HR-HPV assays in different cytology subgroups (n = 172) [file 12935_2020_1512_MOESM3_ESM.doc]

**Table S2. Disagreement between PCR-RDB HPV genotyping and Cervista® HR-HPV assays in different cytology subgroups (n=172)**

| **Group** | **Cytology** | | | **HPV sequencing** | | **Total** |
| --- | --- | --- | --- | --- | --- | --- |
|  | **NILM** | **LSIL** | **HSIL** | **negative** | **positive** |  |
| PCR-RDB (+) &Cervista® (-) | 84 | 9 | 1 | 65 | 29 | 94 |
| PCR-RDB (-) &Cervista® (+) | 70 | 6 | 2 | 62 | 16 | 78 |
| PCR-RDB A5/A6 (+) &Cervista® A5/A6 (-) | 15 | 3 | 0 | 13 | 5 | 18 |
| PCR-RDB A5/A6 (-) &Cervista® A5/A6 (+) | 18 | 4 | 1 | 19 | 4 | 23 |
| PCR-RDB A7 (+) &Cervista® A7 (-) | 38 | 4 | 1 | 31 | 12 | 43 |
| PCR-RDB A7 (-) &Cervista® A7 (+) | 30 | 1 | 0 | 25 | 6 | 31 |
| PCR-RDB A9 (+) &Cervista® A9 (-) | 52 | 7 | 1 | 41 | 19 | 60 |
| PCR-RDB A9 (-) &Cervista® A9 (+) | 36 | 4 | 2 | 32 | 10 | 42 |

**Notes:** PCR-RDB (+), PCR-RDB HPV genotyping assay any HR-HPV types positive; PCR-RDB (-), PCR-RDB HPV genotyping assay no any HR-HPV types positive; Cervista® (+), Cervista® HR-HPV assay any groups positive; Cervista® (-), Cervista® HR-HPV assay all groups negative; PCR-RDB A5/A6, including HPV -51, -56 and -66; PCR-RDB A7, including HPV -18, -39, -45, -59 and -68; PCR-RDB A9, including HPV -16, -31, -33, -35, -52 and -58.
